# The first six years of meta-research at *PLOS Biology*

**DOI:** 10.1371/journal.pbio.3001553

**Published:** 2022-01-31

**Authors:** Roland G. Roberts, on behalf of the PLOS Biology Staff Editors

**Affiliations:** Public Library of Science, San Francisco, California, United States of America and Cambridge, United Kingdom

## Abstract

Meta-research involves the interrogation of every stage of the research lifecycle, from conception to publication and dissemination. Looking back over the first six years of *PLOS Biology* Meta-Research Articles highlights the important insights that can be obtained from such “research on research”.

*PLOS Biology* has been publishing biological research articles for more than 18 years, but six years ago we decided to “go meta”—to recognise the growing need of the research community to scrutinise and challenge how it operates, in an effort to understand and improve the scientific enterprise. To do so, we launched a new article type, the Meta-Research Article, recruited new members to our editorial board to advise us on meta-research submissions and marked the event with an editorial [1] and a blog post.

Reflecting on how the first six years of this initiative have played out has revealed some interesting trends. One intriguing observation is that the meta-research articles reveal an interest in all stages of the grand cycle of the research process (Fig 1), and that each stage brings its own biases. The research community itself is made up of a non-random selection of people. These individuals then choose to devote their efforts to a non-random series of topics. Those research efforts involve methods that may or may not exclude sources of bias from the authors or elsewhere. The research findings are then written up (or shoved into a “file drawer”) with varying degrees of transparency and reporting detail. The resulting manuscripts are assessed by editors and reviewers who bring their own peccadillos to the table, and then published (or not) in journals of varying perceived status. Finally, the articles are disseminated in a number of ways and used or misused by multiple agents, some of whom use them to assess the worth of the researchers and thereby iteratively shape the researcher population.

**Fig 1:**
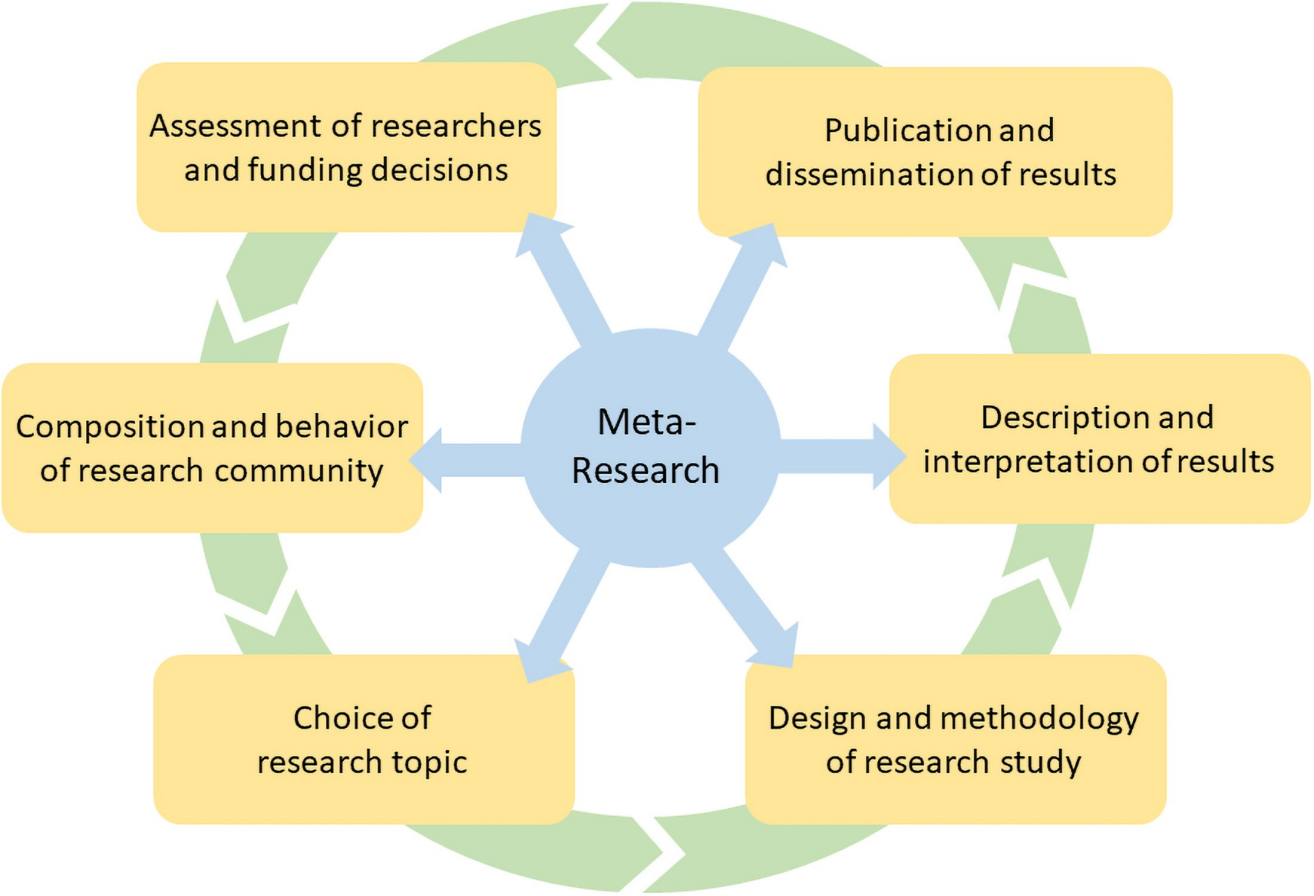
Meta-research interrogates all stages of the research lifecycle.

Each aspect of this cycle can be interrogated by meta-research, and indeed, even within the 43 Meta-Research Articles we have published to date, each aspect is well represented. Six themes that have emerged from this six-year corpus are summarized below, supported by a few choice examples.

1. **The composition and behavior of the research community**. Any human activity is likely to be influenced by the demographics of the relevant community, and scientific research is no exception. *PLOS Biology* studies have explored how factors such as research field, gender and career stage can influence collaboration patterns [2], and how long it will be until men and women are equally represented in various fields [3].
2. **The choice of research topic**. Although all of biology is open for investigation, the topics selected for research are clearly non-random. What influences which literature, organisms, tissues, cells or genes are targeted for study? One study asked what determines the research effort invested in each of the ∼19,000 human genes [4]; another quantified the importance of including non-English language literature in biodiversity surveys [5].
3. **The design and methodology of research studies**. Once any given topic has been selected, the research and subsequent analysis can be performed in myriad different ways, many of which can seriously impact the strength of support for the conclusions. Studies of these choices have included general issues such as the benefits of sample heterogeneity in preclinical work [6] and the effects of statistical model choice on association studies [7], but also the conduct of specific techniques such as functional MRI and RNA sequencing.
4. **The description and interpretation of results**. The distillation of the complex collection of actions that constitute a research project must somehow be compiled into the stereotyped format of a research manuscript. Meta-researchers have systematically examined the role of “spin” in the presentation of scientific results [8] and the way in which microscopy images are prepared for publication [9].
5. **The publication and dissemination of results**. The impact of the study on other workers in the field, on the general public, and on the body of scientific knowledge depends on a set of events that start with a completed manuscript file and pass through posting a preprint, submission to a journal, peer review, revision, publication, press coverage, social media and indexing. Many aspects of this process have been scrutinised, including the misuse of scientific preprints by right-wing social media groups [10] and the effects of article titles on subsequent press coverage [11].
6. **The assessment of researchers and funding decisions**. These published outputs of scientific research, for good or ill, are used by numerous agents to judge the performance of specific researchers and their institutes and to apportion funds accordingly. In turn, these decisions shape the research community of the future. We’ve published papers that address the way that the influence of individual papers can be measured [12] and that propose novel ways of distributing research funding [13].

Over the years, as editors, we have had to decide what does or does not count as “meta-research”. Our understanding of this concept has been shaped by the sheer diversity of manuscripts that have come to us and we anticipate that the next six years will take meta-research in increasingly varied directions. Only by auditing and experimenting with the way we approach discovery can we hope to address some of the issues that pervade research culture today and that threaten to undermine public trust in scientific findings. The launches of initiatives such as the RoRI (Research on Research Institute), the UKRN (UK Reproducibility Network), METRICS (Meta-Research Innovation Center at Stanford) and QUEST (Quality, Ethics, Open Science, Translation), among others, are testament to the growing recognition that science must actively strive to keep its own house in order. The past six years have emphasized the growth and importance of meta-research; “research on research” is clearly here to stay and we look forward to seeing it happen.
